# Correction: Evolutionary Changes in Gene Expression, Coding Sequence and Copy-Number at the *Cyp6g1* Locus Contribute to Resistance to Multiple Insecticides in *Drosophila*

**DOI:** 10.1371/journal.pone.0092490

**Published:** 2014-03-10

**Authors:** The PLOS ONE Staff

Notice of Republication

This paper was republished on February 5, 2014, due to italicization being lost during the production process. The publisher apologizes for the mistake. Please download the paper again to view the corrected version. The originally published, uncorrected article and the republished, corrected article are provided here for reference.

## Supporting Information

File S1 **Originally published, uncorrected article**
(PDF)

File S2 **Republished, corrected article**
(PDF)
