# Supplementary material for: Evolutionary Changes in Gene Expression, Coding Sequence and Copy-Number at the Cyp6g1 Locus Contribute to Resistance to Multiple Insecticides in Drosophila
Source: PLoS One. 2014 Jan 8;9(1):e84879. doi: 10.1371/journal.pone.0084879 (PMC3885650; doi:10.1371/journal.pone.0084879)
Supplement: Table S1 — Primers used to amplify probes for RNA in situ hybridisation and Cyp6g1 orthologs for overexpression studies. (PDF) [file pone.0084879.s002.pdf]

Construct	Primers	Sequence
D. melanogaster & *D. simulans* *in situ* probes	Previously described by Chung *et al*. [11]	
D. willistoni in situ probe	DwilCyp6g1probe-F	5′-ATGGCGTTAACCGAGGCATTGCTG-3′
	DwilCyp6g1probe-F	5′-TCACTGTTTCACTTTCTCATCG-3′
D. virilis in situ probe	DvirCyp6g1probe-F	5′-ATGGCTTTAACGGAGGCGCTTTTTC-3′
	DvirCyp6g1probe-R	5′-TCAGACAGCCTTTTCATCATAAAGTC-3′
Dmel-Cyp6g1 ORF	Dmel6g1-F	5′-CGACAGCGGCCGCATGGTGTTGACCGAGGTC-3′
	Dmel6g1-R	5′-GCGATTCTAGATCATTGGAGCGATGGAGC-3′
Dsim-Cyp6g1 ORF	Dsim6g1-F	5′-GACATCGGTACCATGGTGTTGACC-3′
	Dsim6g1-R	5′-TGCGATACTAGTTCATTGGATCGA-3′
Dvir-Cyp6g1a ORF	Dvir6g1a-F	5′-AGATCTATGGCTTTAACGGAGGCGCTT-3′
	Dvir6g1ab-R	5′-TCTAGATCAGACAGCCTTTTCATCATAAAG-3′
Dvir-Cyp6g1b ORF	Dvir6g1bc-F	5′-CTCGAGATGGCTTTGACGGAGGCGCTC-3′
	Dvir6g1ab-R	5′-TCTAGATCAGACAGCCTTTTCATCATAAAG-3′
Dwil-Cyp6g1 ORF	Dwil6g1-F	5′-GGTACCATGGCGTTAACCGAGGTATTG-3′
	Dwil6g1-R	5′-TCTAGATCACTGTTTCACTTTCTCATCGTA-3′
