# Supplementary material for: Evolutionary Changes in Gene Expression, Coding Sequence and Copy-Number at the Cyp6g1 Locus Contribute to Resistance to Multiple Insecticides in Drosophila
Source: PLoS One. 2014 Jan 8;9(1):e84879. doi: 10.1371/journal.pone.0084879 (PMC3885650; doi:10.1371/journal.pone.0084879)
Supplement: Table S2 — Pairwise amino acid identity (%) between CYP6G1 orthologs. (PDF) [file pone.0084879.s003.pdf]

	Dmel-CYP6G1	Dsim-CYP6G1	Dwil-CYP6G1	Dvir-CYP6G1A	Dvir-CYP6G1B
Dmel-CYP6G1	—	96	75	70	71
Dsim-CYP6G1		—	75	72	72
Dwil-CYP6G1			—	72	70
Dvir-CYP6G1A				—	94
Dvir-CYP6G1B					—
