# Supplementary material for: Correction: Evolutionary Changes in Gene Expression, Coding Sequence and Copy-Number at the Cyp6g1 Locus Contribute to Resistance to Multiple Insecticides in Drosophila
Source: PLoS One. 2014 Mar 10;9(3):e92490. doi: 10.1371/journal.pone.0092490 (PMC3948946; doi:10.1371/journal.pone.0092490)
Supplement: File S2 — Republished, corrected article (PDF) [file pone.0092490.s002.pdf]

OPEN ACCESS Freely available online

PLOS | ONE

Evolutionary Changes in Gene Expression, Coding Sequence and Copy-Number at the *Cyp6g1* Locus Contribute to Resistance to Multiple Insecticides in *Drosophila*

Thomas W. R. Harrop[1], Tamar Sztal[1,2], Christopher Lumb[1], Robert T. Good[1], Phillip J. Daborn[1], Philip Batterham[1]*, Henry Chung[1,3]*

1 Department of Genetics, Bio21 Molecular Science and Biotechnology Institute, The University of Melbourne, Melbourne, Victoria, Australia, 2 School of Biological Sciences, Monash University, Melbourne, Victoria, Australia, 3 Howard Hughes Medical Institute and Laboratory of Molecular Biology, University of Wisconsin, Madison, Wisconsin, United States of America

Abstract

Widespread use of insecticides has led to insecticide resistance in many populations of insects. In some populations, resistance has evolved to multiple pesticides. In *Drosophila melanogaster*, resistance to multiple classes of insecticide is due to the overexpression of a single cytochrome P450 gene, *Cyp6g1*. Overexpression of *Cyp6g1* appears to have evolved in parallel in *Drosophila simulans*, a sibling species of *D. melanogaster*, where it is also associated with insecticide resistance. However, it is not known whether the ability of the CYP6G1 enzyme to provide resistance to multiple insecticides evolved recently in *D. melanogaster* or if this function is present in all *Drosophila* species. Here we show that duplication of the *Cyp6g1* gene occurred at least four times during the evolution of different *Drosophila* species, and the ability of CYP6G1 to confer resistance to multiple insecticides exists in *D. melanogaster* and *D. simulans* but not in *Drosophila willistoni* or *Drosophila virilis*. In *D. virilis*, which has multiple copies of *Cyp6g1*, one copy confers resistance to DDT and another to nitenpyram, suggesting that the divergence of protein sequence between copies subsequent to the duplication affected the activity of the enzyme. All orthologs tested conferred resistance to one or more insecticides, suggesting that CYP6G1 had the capacity to provide resistance to anthropogenic chemicals before they existed. Finally, we show that expression of *Cyp6g1* in the Malpighian tubules, which contributes to DDT resistance in *D. melanogaster*, is specific to the *D. melanogaster*–*D. simulans* lineage. Our results suggest that a combination of gene duplication, regulatory changes and protein coding changes has taken place at the *Cyp6g1* locus during evolution and this locus may play a role in providing resistance to different environmental toxins in different *Drosophila* species.

Citation: Harrop TWR, Sztal T, Lumb C, Good RT, Daborn PJ, et al. (2014) Evolutionary Changes in Gene Expression, Coding Sequence and Copy-Number at the *Cyp6g1* Locus Contribute to Resistance to Multiple Insecticides in *Drosophila*. PLoS ONE 9(1): e84879. doi:10.1371/journal.pone.0084879

Editor: Claude Wicker-Thomas, CNRS, France

Received October 10, 2013; **Accepted** November 28, 2013; **Published** January 8, 2014

Copyright: © 2014 Harrop et al. This is an open-access article distributed under the terms of the Creative Commons Attribution License, which permits unrestricted use, distribution, and reproduction in any medium, provided the original author and source are credited.

Funding: Australian Research Council for funding support. The funders had no role in study design, data collection and analysis, decision to publish, or preparation of the manuscript.

Competing Interests: The authors have declared that no competing interests exist.

* E-mail: p.batterham@unimelb.edu.au (PB); hwchung@wisc.edu (HC)

Introduction

Different mechanisms have evolved that allow organisms to avoid or detoxify harmful chemicals found in their environment or food source. One possible mechanism is a change in a detoxification enzyme that confers a novel metabolic activity, such as the glycine to aspartic acid replacement in the active site of carboxylesterase E3 in the Australian sheep blowfly, which allows the mutant form of the enzyme to hydrolyse the organophosphate insecticide diazinon [1]. A more common mechanism is increased transcription of detoxification enzymes such as cytochrome P450s (P450s) [2], glutathione *S*-transferases [3], or esterases [4,5], either by an increase in the copy number of detoxification genes or by the overexpression of a particular gene. In insects, many examples of insecticide resistance that are caused by or associated with increased transcriptional output of P450s have been documented. For example, overexpression of *Cyp6p3* in field-caught *Anopheles gambiae* is associated with resistance to the insecticide permethrin [6], and gain of *Cyp6bq9* expression in the brain of *Tribolium castaneum* is responsible for deltamethrin resistance [7], while in the aphid *Myzus persicae*, an increase in copy number of the *Cyp6cy3* gene is associated with resistance to neonicotinoid insecticides [8].

Rst(2)DDT, a locus in *D. melanogaster* that is associated with resistance to multiple classes of insecticides in some strains, was mapped to a small region on chromosome 2R containing several P450s. Of these, a single P450 gene, *Cyp6g1*, is overexpressed in resistant strains. There are no amino acid differences in the proteins encoded by the resistant and susceptible alleles of this gene, consistent with evidence that suggests resistance evolved very recently [9,10]. The insertion of the long terminal repeat (LTR) of an *Accord* transposable element into the 5′ region of *Cyp6g1* is responsible for overexpression in the resistant strains, and the overexpression is specific to tissues associated with the metabolism of xenobiotics—the midgut, fat body and Malpighian tubules [11].

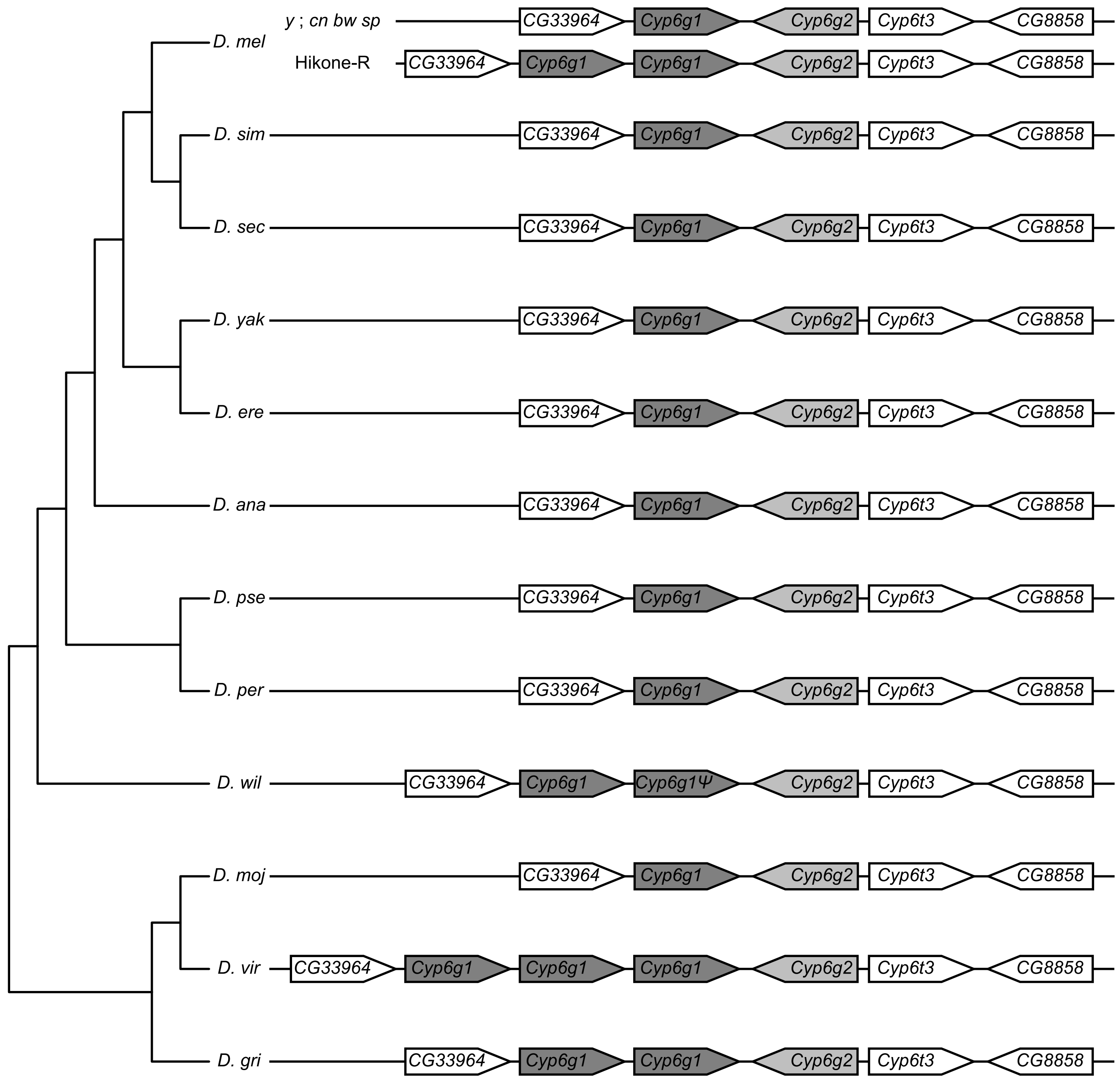

Figure 1. *Cyp6g1* and *Cyp6g2* copy number in twelve *Drosophila* species. *Cyp6g1* is duplicated in *D. willistoni*, *D. grimshawi* and some strains of *D. melanogster*, and triplicated in *D. virilis*. The third copy of *Cyp6g1* in the strain of *D. virilis* used for this study has an inactivating mutation, but this mutation is not present in the sequenced strain, so it is not formally a pseudogene. Comparison with the phylogeny of the species suggests that multiple independent duplication events occurred (cladogram inferred from Stark *et al.* [21]). In contrast, *Cyp6g2* has 1:1 orthologs in all twelve *Drosophila* species analyzed.
doi:10.1371/journal.pone.0084879.g001

A complex, adaptive allelic series exists at this locus, including gene copy-number variation and the insertion of various transposable elements, and each step towards the most derived allele is associated with higher transcription of *Cyp6g1* and increased resistance to dichlorodiphenyltrichloroethane (DDT) [12]. The importance of this gene in adaptation is further highlighted by parallel evolution in the closely related species *Drosophila simulans*. A *Doc* transposable element insertion 5′ to the *Cyp6g1* gene has been identified in *D. simulans* populations. Similar to the *Accord* LTR in *D. melanogaster*, the *Doc* element is associated with a selective sweep, overexpression of *Cyp6g1* and DDT resistance [13]. The constitutive overexpression of *Cyp6g1* is a significant adaptive response, as exposure to insecticides results in very little induction of transcription of *Cyp* genes [14].

Overexpression of *Cyp6g1* in the metabolic tissues provides an example of how increased expression of a single gene can confer resistance to a wide variety of xenobiotics with different chemical structures [11,15]. Because this resistance occurs without changes to the protein sequence, this suggests that the capability of the CYP6G1 protein to provide resistance to various classes of chemicals is present in all *D. melanogaster* strains, and may be an ancestral feature of the protein. If so, then many other insects

might also have the potential to become resistant to the same classes of chemicals by overexpression of *Cyp6g1*. In contrast, if the ability to confer resistance to chemicals is only encoded by *D. melanogaster Cyp6g1* and not by *Cyp6g1* orthologs in closely related species, this would suggest that the potential for broad-spectrum resistance is a derived capacity of CYP6G1 originating from amino acid changes in the *D. melanogaster* lineage. Distinguishing these two possibilities may lead to a better understanding of how resistance to multiple classes of insecticides evolves, as well as contribute to pest management strategies.

Here, we address this question by examining the *Cyp6g1* gene in four *Drosophila* species that last had a common ancestor approximately 40 million years ago [16]. The contribution of regulatory and coding sequence adaptation to the evolution of insecticide resistance was studied by comparing the expression and resistance phenotypes associated with *Cyp6g1* orthologs. Transgenic expression of the *Cyp6g1* orthologs in a consistent genetic background was used to determine whether changes in the coding sequences result in different abilities to provide resistance to xenobiotics, and whether the resistance potential of *Cyp6g1* only exists in *D. melanogaster* or is found in other members of the genus.

Results

Gene duplication of Cyp6g1 has occurred multiple times in the Drosophila genus

As the number of P450s varies between genomes [17,18], and the copy number of *Cyp6g1* is polymorphic in *D. melanogaster* [12], the presence or absence and copy number of *Cyp6g1* in 12 *Drosophila* species with complete reference genomes was investigated [19]. This was accomplished using searches in FlyBase [20] and manual annotation. Orthologs were found in other sequenced *Drosophila* species using the predicted amino acid sequence of CYP6G1 from the sequenced *y; cn bw sp* strain of *D. melanogaster* as a query in BLASTp searches. In the genomes of *D. simulans*, *Drosophila sechellia*, *Drosophila yakuba*, *Drosophila erecta*, *Drosophila annanassae*, *Drosophila pseudoobscura*, *Drosophila persimilis* and *Drosophila mojavensis*, *Cyp6g1* was found as a single copy, as in the *D. melanogaster y; cn bw sp* genome. In *Drosophila willistoni* and *Drosophila grimshawi*, *Cyp6g1* duplications were found, although one copy in *D. willistoni* is truncated and presumably a pseudogene. In *Drosophila virilis*, three copies of *Cyp6g1* were found (**Fig. 1**). In contrast, the closest paralog of *Cyp6g1* in *D. melanogaster*, *Cyp6g2*, exists as a single copy in all 12 *Drosophila* genomes examined. Manual annotation and analysis of the genomic regions surrounding the *Cyp6g1* locus showed conserved microsynteny in all 12 *Drosophila* species (**Fig. 1**).

There are two possible explanations for the multiple copies of *Cyp6g1* in different lineages: either there were independent gene duplication events that occurred in each species that has more than one copy of *Cyp6g1*, or the common ancestor of all twelve species had multiple copies of *Cyp6g1*, but the species with only one copy have independently lost one or more of the ancestral copies. To distinguish between these possibilities, an unrooted neighbour-joining tree of the *Cyp6g1* orthologs from the 12 *Drosophila* genomes was plotted (**Fig. 2**). In the species where there are multiple copies of *Cyp6g1*, the paralogs cluster with each other rather than with *Cyp6g1* orthologs from other species. Considered with the phylogeny of the species [21], this indicates that gene amplification events in each lineage occurred independently, suggesting that some selective advantage to multiple copies of *Cyp6g1* may have existed during the evolution of these species.

Transgenic overexpression of Cyp6g1 orthlogs from different Drosophila species does not produce similar resistance profiles

Transgenic overexpression of *D. melanogaster Cyp6g1* (*Dmel-Cyp6g1*) in the midgut, Malpighian tubules and fat body confers resistance to four different insecticides (DDT, lufenuron, nitenpyram and diazinon) with very different chemical structures [11,15]. When expressed in tobacco cell culture, Dmel-CYP6G1 is able to perform hydroxylation of imidacloprid and dechlorination of DDT—two different chemical reactions on two chemically different insecticides [22]. This indicates that Dmel-CYP6G1 is a P450 with broad substrate specificity.

In order to investigate the functional divergence of CYP6G1, and to determine whether the capacity of Dmel-CYP6G1 to provide resistance to a range of chemicals was ancestral to the different *Drosophila* species or arose in the *D. melanogaster* lineage, orthologs from *D. melanogaster* (*Dmel-Cyp6g1*, cloned from the single-copy *y; cn bw sp* strain), *D. simulans* (*Dsim-Cyp6g1*), *D. willistoni* (*Dwil-Cyp6g1*) and the two expressed paralogs from *D. virilis* (*Dvir-Cyp6g1a* and *Dvir-Cyp6g1b*) were cloned into a specific transgene insertion site in a consistent genetic background (the 86Fb strain) in *D. melanogaster*. The third copy of *Cyp6g1* (*Dvir-Cyp6g1c*) from *D. virilis* was not detected by RT-PCR in several life stages and had an inactivating mutation in the strain used for this study, which is not present in the sequenced strain (data not shown). The use of a defined transgene insertion site allowed us to compare the capability of each ortholog to confer resistance directly, without confounding position effects [23]. The 5′*Cyp6g1*HR-3a-GAL4 driver was used to overexpress the orthologs in the midgut, Malpighian tubules and fat body, an approach which has been validated for testing the resistance potential of genes from distantly related insects [24].

Transgenic flies expressing each ortholog were exposed to three different insecticides (DDT, nitenpyram and dicyclanil) to determine whether overexpression conferred resistance (i.e. increased survival) compared to the background strain (**Fig. 3A**). Overexpression of *Dmel-Cyp6g1* or *Dsim-Cyp6g1* resulted in increased survival on DDT, nitenpyram and dicyclanil. *Cyp6g1* from *D. simulans* and *D. melanogaster* produce qualitatively identical responses under these conditions, but the functions of all the other orthologs were divergent. *Dwil-Cyp6g1* conferred resistance only to dicyclanil. Of the two orthologs from *D. virilis*, *Cyp6g1a* conferred resistance to DDT, and *Cyp6g1b* conferred resistance to nitenpyram (**Fig. 3A**). Although the survival of flies expressing *Dvir-Cyp6g1a* on dicyclanil was statistically higher than control flies, the increase was only 1.01–1.10 fold, suggesting that the resistance is not biologically relevant. The functions of the two copies of *Cyp6g1* from *D. virilis* were qualitatively different despite the 94% identity of their amino acid sequences (**Fig. S1**).

Tissue-specific expression of Cyp6g1 has diverged in the Drosophila genus

The differences in the range of chemicals to which the *Cyp6g1* orthologs from different *Drosophila* species provide resistance when overexpressed raise other questions. Are the *Cyp6g1* orthologs expressed in the same tissues as *Dmel-Cyp6g1* or do they have restricted expression patterns in specialized tissues, as is the case for *Cyp6g2*, which is specifically expressed in the corpus allatum [17]? To investigate this, RNA *in situ* hybridization with probes directed against *Dsim-Cyp6g1*, *Dwil-Cyp6g1* and *Dvir-Cyp6g1* was performed on third instar larvae of these species. Because *Dvir-Cyp6g1a* and *Dvir-Cyp6g1b* are very similar at the nucleotide level, it

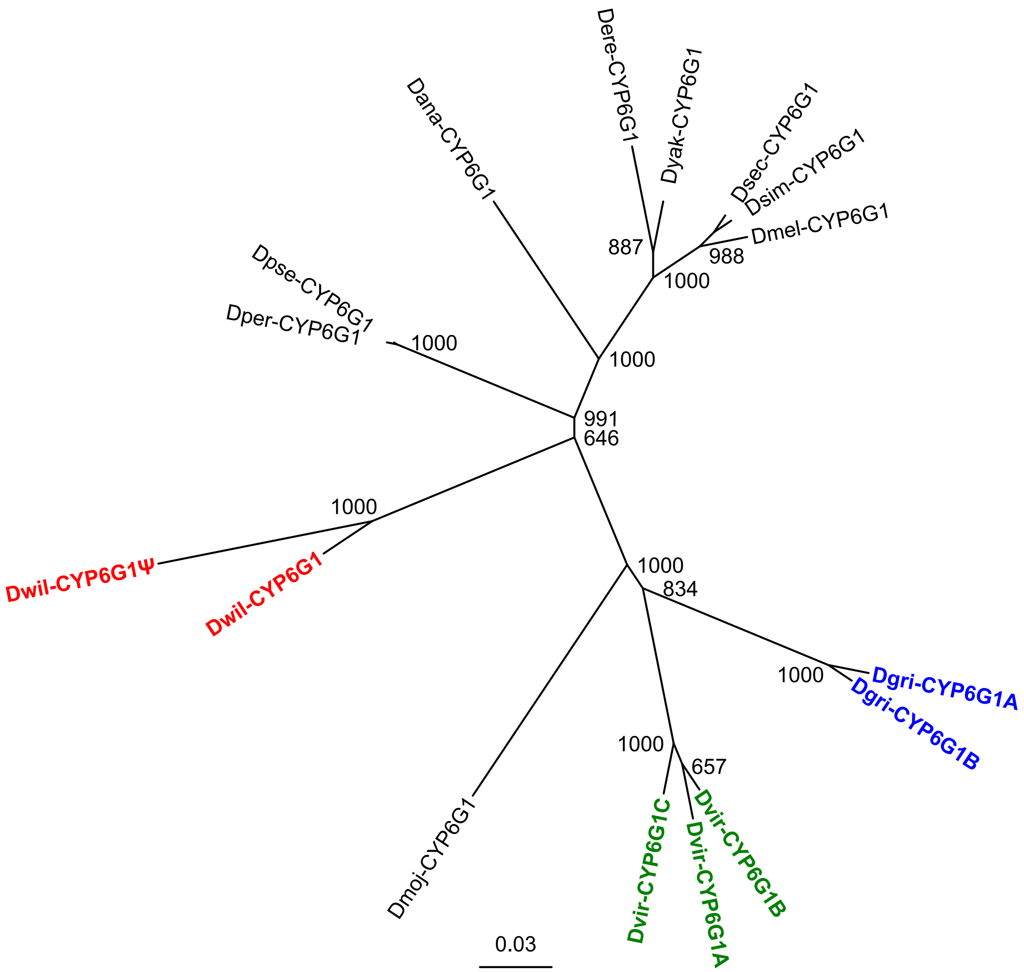

Figure 2. Unrooted neighbour-joining tree of predicted CYP6G1 amino acid sequences from twelve *Drosophila* species. The node labels show bootstrap values from 1000 iterations. Paralogs labelled in the same colour are from the same species. The clustering of paralogs from the same species rather than of orthologs between species supports the hypothesis that the duplications and triplications occurred independently in the separate lineages.
doi:10.1371/journal.pone.0084879.g002

was not possible to design probes that discriminate between the two copies.

The expression of *Dmel-Cyp6g1* has been previously described. Expression in the midgut, Malpighian tubules and fat body is controlled by two distinct tissue-specific enhancers, one that drives expression in the Malpighian tubules and another that drives expression in the midgut and fat body [11,25]. mRNA for the *Cyp6g1* orthologs from all four species was detected in the midgut. However, Malpighian tubule expression as reported in *D. melanogaster* was detected in *D. simulans* but not in *D. willistoni* or *D. virilis*. Fat body expression was detected in all species except *D. willistoni* (**Fig. 4**). These results indicate that gene expression of *Cyp6g1* has evolved in the *Drosophila* genus, and expression of *Cyp6g1* in the Malpighian tubules is restricted to the *D. melanogaster–D. simulans* lineage.

Discussion

In this paper, evidence that *Cyp6g1* is duplicated at least four times in the *Drosophila* genus is presented (**Fig. 1**). Based on phylogenetic analysis of the protein sequences, it can be concluded that the duplications were independent events (**Fig. 2**). There is strong evidence that the duplication in *D. melanogaster* occurred very recently [12]. In contrast, *Cyp6g2*, which is expressed in the corpus allatum in *D. melanogaster* [17], has a 1:1 ortholog in all 12 *Drosophila* species. This supports the inference that across the different *Drosophila* species, there may be selection for increased *Cyp6g1* copy number, presumably via selection for an increased amount of gene product.

In order to determine whether the potential for Dmel-CYP6G1 to provide resistance to insecticides is ancestral, the resistance conferred by overexpressing *Cyp6g1* orthologs from four different species was tested. Similar to *Dmel-Cyp6g1*, overexpression of *Dsim-Cyp6g1* conferred resistance to the three insecticides tested (DDT, nitenpyram and dicyclanil), which corroborates the report of parallel evolution in *D. melanogaster* and *D. simulans* at the *Cyp6g1* locus [13]. Although metabolism of the insecticides was not tested and the metabolic detoxification of insecticides by *Cyp6g1* is not well understood [22], and some orthologs may have the ability to confer resistance to insecticides that were not tested, these results suggest that Dsim-CYP6G1 has metabolic ability and broad substrate specificity similar to Dmel-CYP6G1. In contrast, overexpression of *Dwil-Cyp6g1* only conferred resistance to one of the three insecticides tested (dicyclanil), and the two expressed copies of *Cyp6g1* in *D. virilis*, *Dvir-Cyp6g1a* and *Dvir-Cyp6g1b*,

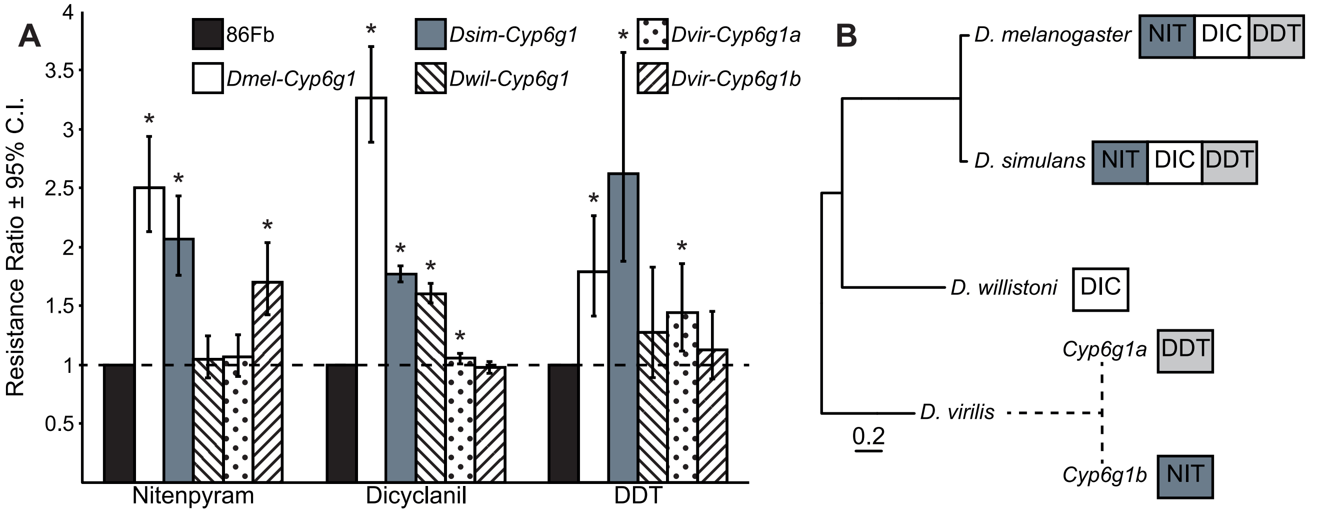

Figure 3. Overexpression of different *Cyp6g1* orthologs confers resistance to different insecticides. A) Changes in survival following exposure to three classes of insecticide by expression of *Cyp6g1* orthologs from *D. melanogaster*, *D. simulans*, *D. willistoni* and *D. virilis* in a consistent genetic background. Resistance ratio (RR) was calculated by comparing the concentration of insecticide that killed 50% of insects (the LC_{50}) between the line expressing the ortholog and the background strain, 86Fb, which was genetically identical except for the absence of the *Cyp6g1* construct. Results marked with an asterisk were statistically significant ($p<0.05$). Orthologs from *D. melanogaster* and *D. simulans* were functionally identical at a qualitative level, both providing resistance to all three chemicals, but the resistance profile varied between the other three orthologs. **B**) Comparison of the potential of the CYP6G1 orthologs to cause resistance when overexpressed. *D. melanogaster* and *D. simulans* orthologs cause resistance to a range of chemicals, whilst the ortholog from *D. willistoni* and the two paralogs from *D. virilis* only conferred resistance to one of the chemicals tested. These results suggest that adaptation of the protein has occurred repeatedly in *Drosophila*. The scale bar indicates the number of substitutions per four-fold degenerate site in the genomes of the species (inferred from Stark *et al.* [21]).
doi:10.1371/journal.pone.0084879.g003

conferred resistance to a narrow range of insecticides compared to *Dmel-Cyp6g1* and *Dsim-Cyp6g1*, with *Dvir-Cyp6g1a* able to confer resistance only to DDT and *Dvir-Cyp6g1b* able to confer resistance only to nitenpyram (**Fig. 3B**). The potential of CYP6G1 to cause resistance when overexpressed has changed frequently and therefore the phylogenetic signal is insufficient to distinguish whether the ancestral CYP6G1 enzyme had the capacity to provide resistance to a broad or limited range of chemicals.

In *D. melanogaster*, transgenic overexpression of *Cyp6g1* in the Malpighian tubules alone results in insecticide resistance, and tissue-specific RNAi knockdown of *Cyp6g1* in the Malpighian tubules results in lower survival on DDT, whilst knockdown in the fat body and central nervous system do not affect survival [26]. Ubiquitous RNAi knockdown of *Cyp6g1* does not cause lethality or observable developmental effects, suggesting that *Cyp6g1* is not involved in development, unlike other P450s tested including *Cyp6g2* [17]. The expression studies indicate that the tissue-specific expression of *Cyp6g1* has evolved in the four species tested. *Cyp6g1* is not expressed in the Malpighian tubules of *D. willistoni* or *D. virilis*. Given that expression of *Cyp6g1* in *D. melanogaster* is controlled by two different enhancers, one for the Malpighian tubules and one for the midgut and fat body, this suggests modularity in the evolution of *Cyp6g1* expression in the different species. Notably, the *Cyp6g1* orthologs from the two species where *Cyp6g1* is expressed in the Malpighian tubules, *D. melanogaster* and *D. simulans*, provide resistance to a range of insecticides. The results presented here do not distinguish whether capacity to provide resistance to a range of insecticides or expression in the Malpighian tubules evolved first in these two species. However, a combination of Malpighian tubule expression and the capacity to provide resistance to a range of insecticides, as observed in *D. melanogaster* and *D. simulans* strains without transposable element insertions, may have conferred a low level of resistance, even before *Cyp6g1* expression was boosted by transposable element insertion, leading to high level resistance to a broad range of insecticides.

The extent to which an enzyme metabolises an insecticide may vary over a continuum. The comparisons between orthologs were based on a threshold of resistance, which is expected to be related to the amount of metabolic activity the enzyme has towards the insecticide, but this was not measured. When genes that are associated with metabolism-based insecticide resistance in pest species are expressed in *D. melanogaster*, the levels of resistance are generally lower than in the pest species [24]. In this study, the amount of transcript produced for each ortholog was not measured and antibodies for quantification of the proteins are not available. Previous studies have indicated that a high level of transcript is produced using this system, but even if the amount of transcript was controlled across the different transgenic lines, effects such as codon bias and translational efficiency might still result in differing amounts of functional protein, and other factors may be lacking in *D. melanogaster* that are required for the enzyme to function optimally [15]. Nevertheless, the fact that resistance was conferred to at least one of the insecticides by each ortholog indicates that a significant amount of functional protein was produced, and supports the hypothesis that the potential for the *Cyp6g1* gene to provide resistance existed in the last common ancestor of the four *Drosophila* species, approximately 40 million years ago. This implies that the ability of *Cyp6g1* to provide resistance to anthropogenic insecticides when overexpressed predates the existence of the compounds, so this property may be a side effect of adaptation to detoxify allelochemicals present in the food source. Previous evidence indicates that selection with plant allelochemicals affects tolerance to insecticides in moths, and P450 induction by allelochemicals is correlated with insecticide resistance [27,28].

The different resistance profiles of the orthologs suggest that the chemicals to which the CYP6G1 protein has the potential to cause

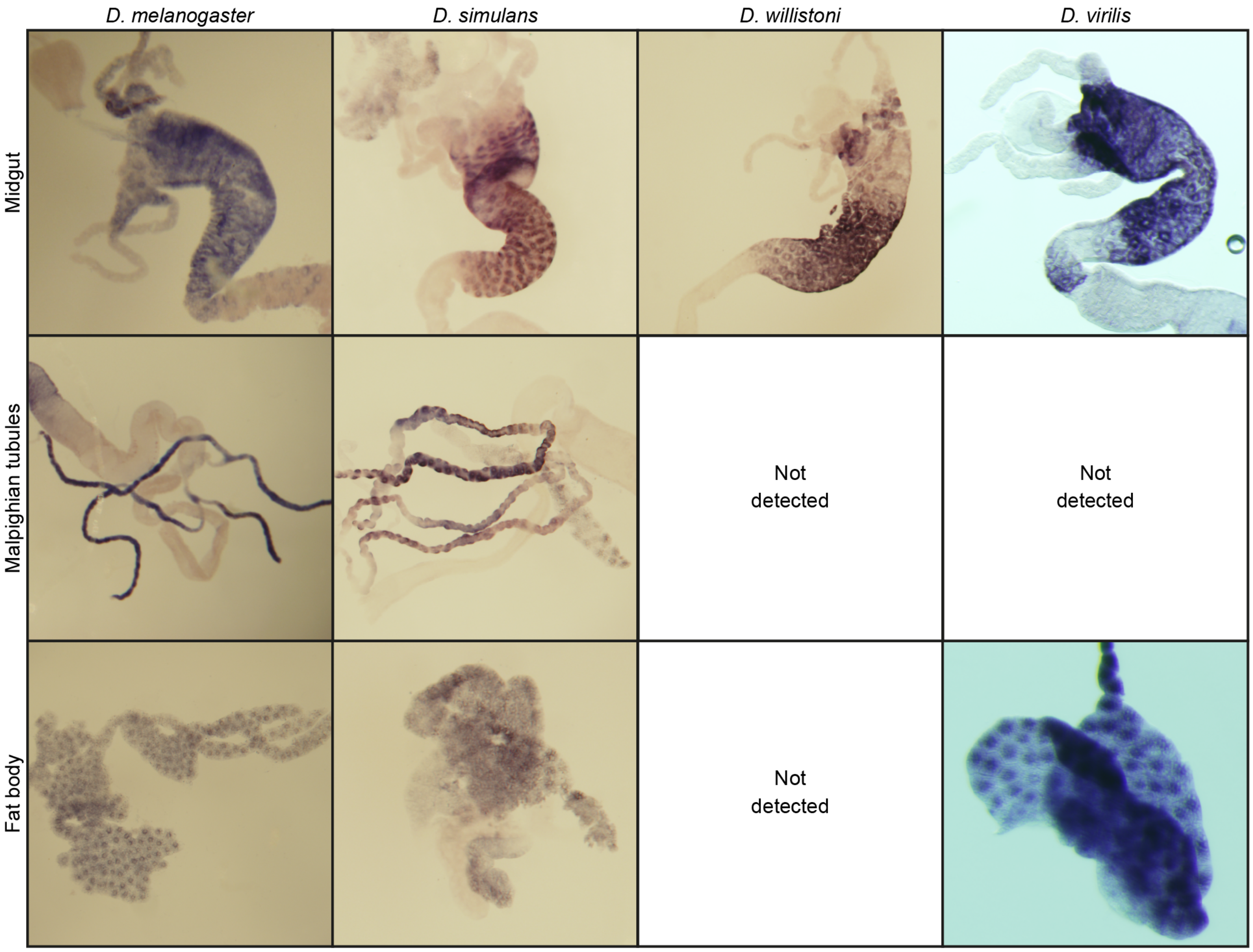

Figure 4. RNA *in situ* hybridization experiments for *Cyp6g1* in the midgut, Malpighian tubules and fat body of third instar larvae of *D. melanogaster*, *D. simulans*, *D. willistoni* and *D. virilis*. Expression was observed in the midgut in all four species. Fat body expression was observed in all species except *D. willistoni*, and Malpighian tubule expression was not detected in *D. willistoni* or *D. virilis*, the two species from which orthologs only conferred resistance to one of the insecticides tested. The expression of *Cyp6g1* in *D. melanogaster* has been described previously but is included here for comparison with the species tested in this study [11].
doi:10.1371/journal.pone.0084879.g004

resistance are not the same throughout the *Drosophila* lineage, and it may have evolved to detoxify allelochemicals specific to the habitat of each species. This possibility is highlighted by the duplication of *Cyp6g1* in *D. virilis*, which gave rise to two enzymes with different capacities for resistance. Comparison of the predicted amino acid sequences of the orthologs did not reveal differences that correlate with the differences in resistance when overexpressed (**Table S2, Fig. S1**). Although a homology model for CYP6G1 has been produced [29], there is not enough information about the relationship between P450 structure and function to allow prediction of metabolic capacity based on primary sequence [30,31], and the lack of functional validation of the model means that these results must be interpreted with caution.

The data presented here suggest that the evolution of resistance to different insecticides involves adaptive changes in both tissue-specific gene expression and coding sequence. These findings may have implications for strategies to combat metabolic insecticide resistance in the field, and contribute to the understanding of the mechanisms by which different evolutionary changes lead to resistance to multiple insecticides. If the ability of P450s to provide resistance to anthropogenic insecticides is a side effect of their evolution to detoxify host xenobiotics, then supposedly naïve insect populations may possess an enzyme activity that can provide resistance to novel insecticides, despite not being optimised for their metabolism. In this case, a regulatory change to increase P450 expression in the appropriate tissues, such as a transposable element insertion [10,13], would be enough to confer resistance to an insect population.

Materials and Methods

Annotation of orthologs and phylogenetic analyses

The genomic region surrounding *Cyp6g1* in each of the 12 *Drosophila* species was manually annotated using the Artemis software package [32]. The predicted amino acid sequences of the orthologs were aligned and an unrooted, neighbour-joining tree with 1000 bootstrapping iterations was produced with ClustalX 2.1 [33].

RNA in situ hybridization

RNA *in situ* hybridization was performed as per published protocols [11]. The *D. melanogaster* probe was used for *in situ* hybridization for *D. simulans*. The primers used to design *D. willistoni* and *D. virilis* probes are listed in the **Table S1**.

Overexpression of Cyp6g1 orthologs and insecticide resistance assays

The *Cyp6g1* orthologs were cloned using multiple steps into *pUASTattB* using the *EcoRI* sites and the primers listed in the **Table S1** from cDNA synthesized from a Caribbean isolate of *D. willistoni* Quechua (UCSD stock number 14030-0814.10), a Californian isolate of *D. virilis* (UCSD stock number 15010-1051.00) and a population of *D. simulans*. The UAS-*Cyp6g1* constructs were transformed into the y^1 *M{vas-int.Dm}*ZH-2A w^*; *M{3×P3-RFP.attP}*ZH-86Fb recipient strain (herein referred to as 86Fb), which has a defined integration site on Chromosome III, using the *attP–attB* system and φC31 integrase [23]. Expression of the *Cyp6g1* orthologs was achieved using the *Gal4*–UAS system by crossing males carrying the UAS–*Cyp6g1* constructs to virgin 5′*Cyp6g1*HR-3a females, which express *GAL4* in the midgut, Malpighian tubules and fat body [11]. The background of the progeny was controlled by repeating the cross using 86Fb males instead of UAS–*Cyp6g1* males. 3–8 replicates of twenty 4-day-old, mated, adult female progeny were exposed to concentrations of 1–5 $\mu g \cdot vial^{-1}$ of DDT (Sigma) using 24 hour contact assays in glass scintillation vials [9]. The number of replicates for each cross at each dose was determined by the abundance of adult flies produced by the cross, and no data were excluded from the analysis. Ten replicates of twenty-five first instar larvae were reared on food containing $0.8–4.5\times10^{-4}$ % w/v nitenpyram (Novartis) and five replicates on $0.9–1.6\times10^{-6}$ % w/v dicyclanil (Novartis) and emergence was counted after 15 days. Dosage mortality curves were constructed and LC_{50} values estimated using PriProbit [34] and resistance ratios and confidence intervals were calculated using the method described by Robertson *et al.* [35].

Supporting Information

Figure S1 Multiple alignment of the predicted amino acid sequences of the CYP6G1 orthologs that were transgenically expressed in *D. melanogaster*. There were no changes observed in any of the putatitive P450 functional domains [36], except a serine to phenylalanine substitution at residue 317 in Dsim-CYP6G1, which did not result in any functional differences between Dsim-CYP6G1 and Dmel-CYP6G1 in our experiments.
(EPS)

Table S1 Primers used to amplify probes for RNA *in situ* hybridisation and *Cyp6g1* orthologs for overexpression studies.
(PDF)

Table S2 Pairwise amino acid identity (%) between CYP6G1 orthologs.
(PDF)

Acknowledgments

We thank C. Robin, H. Dufour and D. Loehlin for valuable discussion and feedback on the manuscript, and S.W. McKechnie for *Drosophila* stocks.

Author Contributions

Conceived and designed the experiments: TWRH PJD PB HC. Performed the experiments: TWRH TS CL RTG PJD HC. Analyzed the data: TWRH TS PJD PB HC. Wrote the paper: TWRH TS PB HC.

References

1. Newcomb RD, Campbell PM, Ollis DL, Cheah E, Russell RJ, et al. (1997) A single amino acid substitution converts a carboxylesterase to an organophosphorus hydrolase and confers insecticide resistance on a blowfly. Proceedings of the National Academy of Sciences 94: 7464–7468.
2. Feyereisen R (2005) Insect Cytochrome P450. In: Gilbert LI, Iatrou K, Gill SS, editors. Comprehensive Molecular Insect Science. Comprehensive Molecular Insect Science. Amsterdam: Elsevier B.V., Vol. 5. pp. 1–77. Available: http://www.sciencedirect.com/science/article/pii/B0444519246000491.
3. Low WY, Ng HL, Morton CJ, Parker MW, Batterham P, et al. (2007) Molecular evolution of glutathione *S*-transferases in the genus Drosophila. Genetics 177: 1363–1375. doi:10.1534/genetics.107.075838.
4. Field LM, Devonshire AL, Forde BG (1988) Molecular evidence that insecticide resistance in peach-potato aphids (*Myzus persicae* Sulz.) results from amplification of an esterase gene. Biochem J 251: 309–312.
5. Mouchès C, Pasteur N, Bergé JB, Hyrien O, Raymond M, et al. (1986) Amplification of an esterase gene is responsible for insecticide resistance in a California *Culex* mosquito. Science 233: 778–780.
6. Müller P, Warr E, Stevenson BJ, Pignatelli PM, Morgan JC, et al. (2008) Field-caught permethrin-resistant *Anopheles gambiae* overexpress CYP6P3, a P450 that metabolises pyrethroids. PLoS Genet 4: e1000286. doi:10.1371/journal.pgen.1000286.
7. Zhu F, Parthasarathy R, Bai H, Woithe K, Kaussmann M, et al. (2010) A brain-specific cytochrome P450 responsible for the majority of deltamethrin resistance in the QTC279 strain of *Tribolium castaneum*. Proceedings of the National Academy of Sciences 107: 8557–8562. doi:10.1073/pnas.1000059107.
8. Puinean AM, Foster SP, Oliphant L, Denholm I, Field LM, et al. (2010) Amplification of a cytochrome P450 gene is associated with resistance to neonicotinoid insecticides in the aphid *Myzus persicae*. PLoS Genet 6: e1000999. doi:10.1371/journal.pgen.1000999.
9. Daborn PJ, Boundy S, Yen J, Pittendrigh BR, ffrench-Constant RH (2001) DDT resistance in *Drosophila* correlates with *Cyp6g1* over-expression and confers cross-resistance to the neonicotinoid imidacloprid. Mol Genet Genomics 266: 556–563. doi:10.1007/s004380100531.
10. Daborn PJ, Yen J, Bogwitz MR, Le Goff G, Feil E, et al. (2002) A single p450 allele associated with insecticide resistance in *Drosophila*. Science 297: 2253–2256. doi:10.1126/science.1074170.
11. Chung H, Bogwitz MR, McCart C, Andrianopoulos A, ffrench-Constant RH, et al. (2007) *Cis*-regulatory elements in the *Accord* retrotransposon result in tissue-specific expression of the *Drosophila melanogaster* insecticide resistance gene *Cyp6g1*. Genetics 175: 1071–1077. doi:10.1534/genetics.106.066597.
12. Schmidt JM, Good RT, Appleton B, Sherrard J, Raymant GC, et al. (2010) Copy number variation and transposable elements feature in recent, ongoing adaptation at the *Cyp6g1* locus. PLoS Genet 6: e1000998. doi:10.1371/journal.pgen.1000998.
13. Schlenke T, Begun D (2004) Strong selective sweep associated with a transposon insertion in *Drosophila simulans*. Proceedings of the National Academy of Sciences 101: 1626–1631. doi:10.1073/pnas.0303793101.
14. Willoughby L, Chung H, Lumb C, Robin C, Batterham P, et al. (2006) A comparison of *Drosophila melanogaster* detoxification gene induction responses for six insecticides, caffeine and phenobarbital. Insect Biochemistry and Molecular Biology 36: 934–942. doi:10.1016/j.ibmb.2006.09.004.
15. Daborn PJ, Lumb C, Boey A, Wong W, ffrench-Constant RH, et al. (2007) Evaluating the insecticide resistance potential of eight *Drosophila melanogaster* cytochrome P450 genes by transgenic over-expression. Insect Biochemistry and Molecular Biology 37: 512–519. doi:10.1016/j.ibmb.2007.02.008.
16. Russo CA, Takezaki N, Nei M (1995) Molecular phylogeny and divergence times of Drosophilid species. Molecular Biology and Evolution 12: 391–404.
17. Chung H, Sztal T, Pasricha S, Sridhar M, Batterham P, et al. (2009) Characterization of *Drosophila melanogaster* cytochrome P450 genes. Proceedings of the National Academy of Sciences 106: 5731–5736. doi:10.1073/pnas.0812141106.
18. Thomas JH (2007) Rapid birth-death evolution specific to xenobiotic cytochrome P450 genes in vertebrates. PLoS Genet 3: 720–728. doi:10.1371/journal.pgen.0030067.
19. Clark AG, Eisen MB, Smith DR, Bergman CM, Oliver B, et al. (2007) Evolution of genes and genomes on the *Drosophila* phylogeny. Nature 450: 203–218. doi:10.1038/nature06341.
20. Marygold SJ, Leyland PC, Seal RL, Goodman JL, Thurmond J, et al. (2013) FlyBase: improvements to the bibliography. Nucleic Acids Research 41: D751–D757. doi:10.1093/nar/gks1024.
21. Stark A, Lin MF, Kheradpour P, Pedersen JS, Parts L, et al. (2007) Discovery of functional elements in 12 *Drosophila* genomes using evolutionary signatures. Nature 450: 219–232. doi:10.1038/nature06340.

22. Joußen N, Heckel DG, Haas M, Schuphan I, Schmidt B (2008) Metabolism of imidacloprid and DDT by P450 GYP6G1 expressed in cell cultures of *Nicotiana tabacum* suggests detoxification of these insecticides in *Cyp6g1*-overexpressing strains of *Drosophila melanogaster*, leading to resistance. Pest Management Science 64: 65–73. doi:10.1002/ps.1472.
23. Bischof J, Maeda RK, Hediger M, Karch F, Basler K (2007) An optimized transgenesis system for *Drosophila* using germ-line-specific φC31 integrases. Proceedings of the National Academy of Sciences 104: 3312–3317. doi:10.1073/pnas.0611511104.
24. Daborn PJ, Lumb C, Harrop TWR, Blasetti A, Pasricha S, et al. (2012) Using *Drosophila melanogaster* to validate metabolism-based insecticide resistance from insect pests. Insect Biochemistry and Molecular Biology 42: 918–924. doi:10.1016/j.ibmb.2012.09.003.
25. Chung H, Boey A, Lumb C, Willoughby L, Batterham P, et al. (2011) Induction of a detoxification gene in *Drosophila melanogaster* requires an interaction between tissue specific enhancers and a novel *cis*-regulatory element. Insect Biochemistry and Molecular Biology 41: 863–871. doi:10.1016/j.ibmb.2011.07.002.
26. Yang J, McCart C, Woods DJ, Terhzaz S, Greenwood KG, et al. (2007) A *Drosophila* systems approach to xenobiotic metabolism. Physiol Genomics 30: 223–231. doi:10.1152/physiolgenomics.00018.2007.
27. Li X, Zangerl AR, Schuler MA, Berenbaum MR (2000) Cross-resistance to alpha-cypermethrin after xanthotoxin ingestion in *Helicoverpa zea* (Lepidoptera: Noctuidae). J Econ Entomol 93: 18–25.
28. Zeng RS, Wen Z, Niu G, Schuler MA, Berenbaum MR (2007) Allelochemical induction of cytochrome P450 monooxygenases and amelioration of xenobiotic toxicity in *Helicoverpa zea*. J Chem Ecol 33: 449–461. doi:10.1007/s10886-006-9238-1.
29. Jones RT, Bakker SE, Stone D, Shuttleworth SN, Boundy S, et al. (2010) Homology modelling of *Drosophila* cytochrome P450 enzymes associated with insecticide resistance. Pest Management Science 66: 1106–1115. doi:10.1002/ps.1986.
30. Feyereisen R (2012) Insect CYP Genes and P450 Enzymes. In: Gilbert LI, editor. Insect Molecular Biology and Biochemistry. Amsterdam: Elsevier B.V. pp. 236–316. doi:10.1016/B978-0-12-384747-8.10008-X.
31. Kirischian N, McArthur AG, Jesuthasan C, Krattenmacher B, Wilson JY (2011) Phylogenetic and functional analysis of the vertebrate cytochrome P450 2 family. J Mol Evol 72: 56–71. doi:10.1007/s00239-010-9402-7.
32. Rutherford K, Parkhill J, Crook J, Horsnell T, Rice P, et al. (2000) Artemis: sequence visualization and annotation. Bioinformatics 16: 944–945.
33. Larkin MA, Blackshields G, Brown NP, Chenna R, McGettigan PA, et al. (2007) Clustal W and Clustal X version 2.0. Bioinformatics 23: 2947–2948. doi:10.1093/bioinformatics/btm404.
34. Sakuma M (1998) Probit analysis of preference data. Appl Entomol Zool 33: 339–347.
35. Robertson JL, Savin NE, Preisler HK, Russell RM (2007) Bioassays with Arthropods. 2nd ed. CRC Press.
36. Werck-Reichhart D, Feyereisen R (2000) Cytochromes P450: a success story. Genome Biology 1: REVIEWS3003. doi:10.1186/gb-2000-1-6-reviews3003.
